# Dynamic parent-of-origin effects on small interfering RNA expression in the developing maize endosperm

**DOI:** 10.1186/s12870-014-0192-8

**Published:** 2014-07-24

**Authors:** Mingming Xin, Ruolin Yang, Yingyin Yao, Chuang Ma, Huiru Peng, Qixin Sun, Xiangfeng Wang, Zhongfu Ni

**Affiliations:** 1 State Key Laboratory for Agrobiotechnology, Key Laboratory of Crop Heterosis Utilization (MOE), Beijing Key Laboratory of Crop Genetic Improvement, China Agricultural University, NO.2 Yuanmingyuan Xi Road, Haidian District, Beijing 100193, China; 2 School of Plant Sciences, University of Arizona, 1145 E. South Campus Drive, Tucson 85721-0036, AZ, USA

**Keywords:** siRNA, Imprinting, Maize, Endosperm, GSE52726

## Abstract

**Background:**

In angiosperms, the endosperm plays a crucial placenta-like role in that not only is it necessary for nurturing the embryo, but also regulating embryogenesis through complicated genetic and epigenetic interactions with other seed compartments and is the primary tissue in which genomic imprinting occurs.

**Results:**

We observed a gradual increase of paternal siRNA expression in the early stages of kernels and an expected 2:1 maternal to paternal ratio in 7-DAP endosperm *via* sequencing of small interfering RNA (siRNA) transcriptomes in developing kernels (0, 3 and 5 days after pollination (DAP)) and endosperms (7, 10 and 15 DAP) from the maize B73 and Mo17 reciprocal crosses. Additionally, 460 imprinted siRNA loci were identified in the endosperm, with the majority (456/460, 99.1%) being maternally expressed at 10 DAP. Moreover, 13 out of 29 imprinted genes harbored imprinted siRNA loci within their 2-kb flanking regions, a significant higher frequency than expected based on simulation analysis. Additionally, gene ontology terms of “response to auxin stimulus”, “response to brassinosteroid stimulus” and “regulation of gene expression” were enriched with genes harboring 10-DAP specific siRNAs, whereas those of “nutrient reservoir activity”, “protein localization to vacuole” and “secondary metabolite biosynthetic process” were enriched with genes harboring 15-DAP specific siRNAs.

**Conclusions:**

A subset of siRNAs subjected to imprinted expression pattern in maize developing endosperm, and they are likely correlated with certain imprinted gene expression. Additionally, siRNAs might influence nutrient uptake and allocation processes during maize endosperm development.

## Background

In flowering plants, the two distinct female gametes, the central cell (2n) and the egg cell (1n), are fertilized by two sperm cells (1n), producing the triploid endosperm (2-maternal vs. 1-paternal, 2 m:1p hereafter) and the diploid embryo (1 m:1p), respectively [1]. The genetic balance and molecular interaction between the endosperm, embryo and surrounding maternal tissues are essential to proper seed development [2]-[6]. Although a terminal tissue, the endosperm plays a crucial role by providing nutrients to support embryogenesis and seed germination and coordinate seed development [2]. However, how the expression of the parental genome is regulated and what proportions of the filial genomes contribute to the control of this process during early endosperm development have not been fully explored. Although some studies have indicated that the transcriptional activation of the paternal genome lags behind that of the maternal genome in the seed development during the first few days after pollination (DAP), resulting in an unequal expression of parental genomes [7]-[9], other studies have reported that the parental genomes might be initiated concomitantly after fertilization, even at the 2-to 4-cell stage [10]-[12].

Imprinting refers to allele-specific gene expression that depends on the parent-of-origin manner, a form of unequal contribution by parental genomes primarily occurring in the plant endosperm [13]-[16]. Recent studies in *Arabidopsis thaliana* and *Oryza sativa* (rice) have revealed that a subset of non-coding loci generating small interfering RNAs (siRNAs) are also subject to parental genomic imprinting [17],[18]. For example, the predominant accumulation of PolIV-dependent 24-nt siRNAs was transcribed from the maternal genome after fertilization in *Arabidopsis* seeds of the Columbia and Landsberg erecta reciprocal crosses. The expression of these siRNAs peaked at 4 to 6 DAP, and decreased in later seed developmental stages [17]. In addition, the biogenesis of the 24-nt siRNAs was dependent on the maternal genome dosage in the *Arabidopsis* endosperm of interploidy crosses in which the proportion of the 24-nt siRNAs was 9% greater in the maternal-excess cross than in the paternal-excess cross, likely influencing the expression of *AGAMOUS*-*LIKE* genes and resulting in smaller seeds in the maternal-excess cross compared to the paternal-excess cross [19]. Whereas in the hybrid rice endosperm between *Nipponbare* and *Kitaake*, 31 imprinted siRNA loci were identified at 7 to 8 DAP, including 15 maternally and 16 paternally expressed siRNA loci. This finding indicated that both paternal and maternal siRNAs were subject to imprinting in the rice endosperm, which was different from the strong maternal tendency of siRNA expression in *Arabidopsis*[18], and also, these imprinted 24-nt siRNA loci were enriched in the differential methylation regions (DMRs) in the rice endosperm, similar to the imprinted protein-coding genes [17].

siRNAs were proved to participate in the silencing the paternal allele of *SUPPRESSOR OF drm1 drm2 drm3* (*SDC*), resulting in its maternally specific expression in *Arabidopsis* endosperm, by detecting the *SDC* promoter activity in *pSDC*::*H2B*-*RFP* and wild type (WT) reciprocal crosses and in the WT × *pSDC*::*H2B*-*RFP*(*nrpd2a*) cross [20]. Additionally, siRNAs are also likely involved in the silencing of the paternal allele of *MOP9.5*, as indicated by an allele-specific RT-PCR analysis in the endosperm of WT and *nrpd2a* reciprocal crosses [20]. However, there is no compelling evidence exhibiting a close association between imprinted siRNAs and imprinted genes, although a proportion of the imprinted siRNAs have been reported to be associated with the imprinted gene expression, for instance, four imprinted siRNA loci were found to overlap with differential DMRs proximal to imprinted genes in the rice endosperm and showed opposite parental expression patterns compared to the corresponding imprinted genes [18] In addition, 12 transposable elements surrounding the paternal alleles of maternally expressed genes (MEGs) were targeted by 24-nt siRNAs in pollen and might be responsible for the silencing of the paternal alleles in the *Arabidopsis* endosperm after fertilization [21].

Based on a comprehensive list of single nucleotide polymorphisms (SNPs) detected between the fully assembled Mo17 genome and the reference B73 genome (release 5b.60, http://MaizeSequence.org), we previously identified 290 imprinted protein-coding genes by RNA-Seq analysis in developing maize kernels at 0, 3 and 5 DAP and in endosperms at 7, 10 and 15 DAP [22]. These stages represent the key developmental events that are central to the endosperm’s role as an absorptive structure, including early cell proliferation, compartment differentiation, initiation of starch and storage protein accumulation and rapid fresh weight increases [5],[23]. A gene ontology (GO) analysis of imprinted genes identified 10-DAP-specific MEGs that are involved in maize endosperm nutrient uptake and allocation mediated by the auxin signaling pathway [22].

In this study, to identify imprinted siRNAs and understand the developmental bias of parental siRNA expression in the maize early endosperm, we further sequenced siRNA transcriptomes using the same materials as before at six developmental stages, and observed the early transcriptional activation of paternal siRNAs in the 3- and 5-DAP kernels and identified 460 imprinted siRNA loci in the 7-, 10- and 15-DAP hybrid endosperms. Our data indicate, similar to imprinted genes, that the expression of imprinted siRNA loci changes dynamically in developing endosperms, with the majority of maternally expressed siRNA loci (MESL) being uniquely expressed at 10 DAP. Additionally, imprinted siRNAs were predicted to be significantly associated with imprinted gene expression in maize endosperm in terms of adjacency based on a simulation analysis, and the GO categories of siRNA-associated genes revealed that siRNAs were likely to play a role in key events of endosperm development.

## Results

### SiRNA transcriptome sequencing

We sequenced 12 siRNA samples including the kernels (0, 3 and 5 DAP) and endosperms (7, 10 and 15 DAP) harvested from the maize B73 and Mo17 reciprocal crosses. On average, 13.6 million adaptor-trimmed siRNA reads were produced for each sample, ranging from 18 to 27 nucleotides (nt) in length. The three most abundant classes were 24-, 22- and 21-nt siRNAs, representing approximately 55.3%, 13.4% and 12.6% of the total siRNAs, respectively (Additional file 1: Figure S1). The kernel samples exhibited higher proportions of 24-nt siRNAs (70.7% at 0 DAP, 68.1% at 3 DAP and 64.4% at 5 DAP) compared with the endosperm samples (36.6% at 7 DAP, 50.1% at 10 DAP and 41.8% at 15 DAP), whereas the proportions of 21- and 22-nt siRNAs in the endosperm samples were higher (37.4% at 7 DAP, 26.7% at 10 DAP and 34.9% at 15 DAP) compared with those of the kernel samples (17.4% at 0 DAP, 18.0% at 3 DAP and 21.2% at 5 DAP) (Additional file 1: Figure S1). The siRNA reads of 12 samples were mapped to the B73 reference genome (release 5.6b, http://MaizeSequence.org), and only the perfectly matched reads were retained for further analysis. Approximately 37.6% of the reads were uniquely mapped on the B73 genome, whereas 58.8%, 68.2% and 74.0% of the reads were mapped on the B73 genome, respectively, when maximally 10, 100 and 1,000 multiple matched locations for a read (multi-reads) were allowed (Additional file 2: Table S1).

### Genomic and genic distributions of 24-nt siRNAs

**Figure 1:**
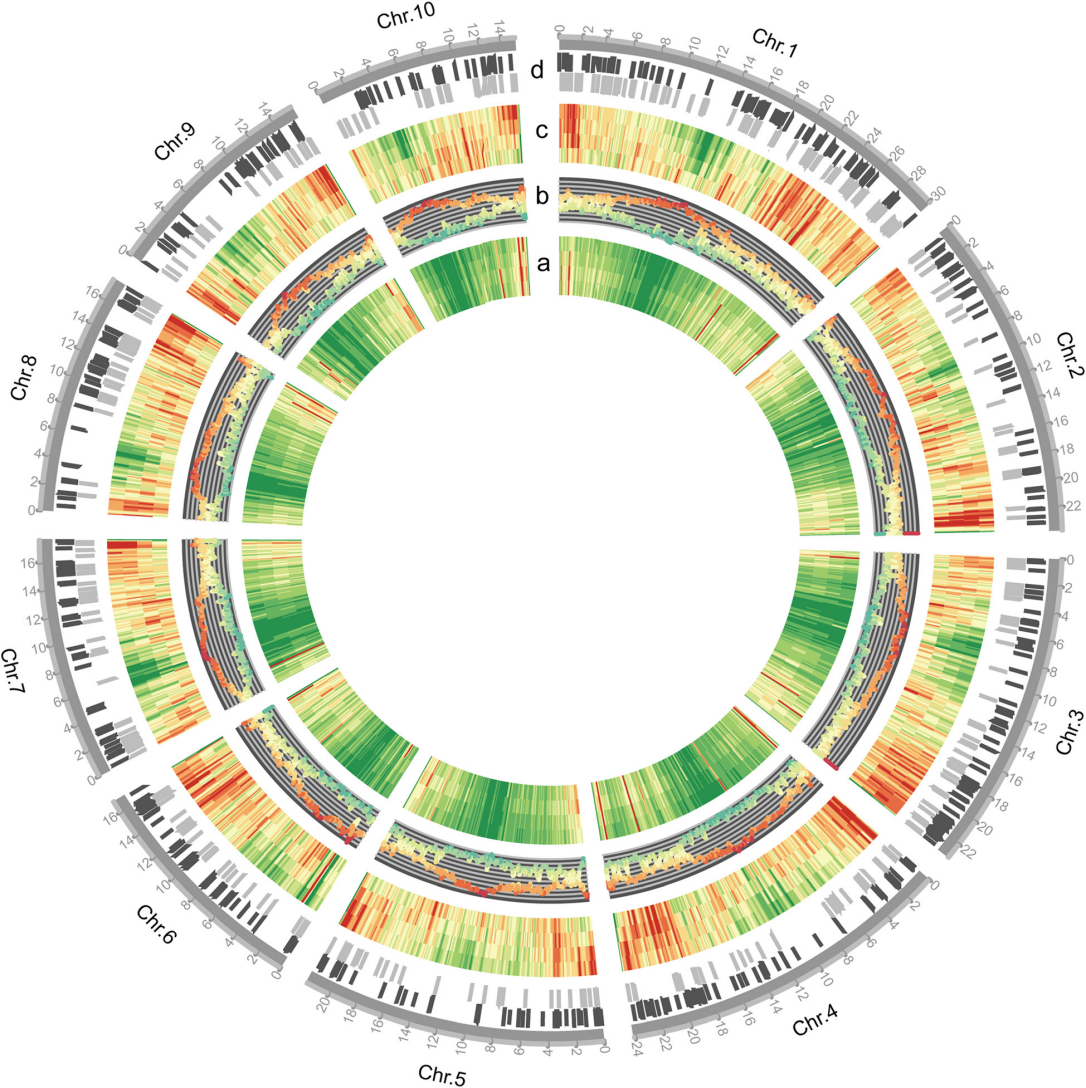
**Chromosomal distribution of siRNA loci and imprinted genes on maize genome.** Lane group a: Chromosomal distribution of siRNA reads abundance. These four lanes indicate differential siRNA abundance along maize chromosomes using perfectly mapped siRNA with unique position, ≤10, ≤100, and ≤1000 positions on maize genome, respectively, from outside to inside. They were preferentially enriched in high-density gene regions and chromosomal end regions but devoid in repeat-rich pericentromeric and centromeric regions. Red: high abundance; Yellow: medium abundance; Green: low abundance. Lane b: Chromosomal distribution of protein-coding genes and repeat sequences identified by Repeat-Mask software, and repeat sequences were highly enriched in centromeric and telomeric regions, which exhibited a negative correlation with gene density along chromosomes. Red: high proportion of repeats; Green: high proportion of genes; Yellow: relatively equal proportion of genes and repeats. Lane group c: Chromosomal distributions of siRNA loci identified by using the reads uniquely mapped, mapped with ≤10, ≤100 and ≤1000 positions respectively, from outside to inside, and the trend is similar to the siRNA abundance distribution. Red: high density; Yellow: moderate density; Green: low density. Lane d: Chromosomal distributions of imprinted genes (light gray) and imprinted siRNA loci (dark gray) on maize genome, which were distributed on all 10 chromosomes.

We identified and classified genomic loci generating siRNAs based on previous methods [24],[25] (Methods), and these siRNA loci were further defined in terms of their dominant siRNA population representing more than 50% of the total siRNAs at a locus. When only using uniquely mapped reads, 416,100 siRNA loci were identified across all ten chromosomes; when multi-reads were included, 644,496, 836,618 and 931,924 siRNA loci were identified with the maximum mapping locations of 10, 100 and 1,000, respectively. The chromosomal distribution demonstrated that siRNA loci were preferentially enriched in high-density gene regions and chromosomal end regions but devoid in repeat-rich pericentromeric and centromeric regions (Figure 1), which is consistent with the observation in maize shoots that only a small proportion of siRNAs are generated from the pericentromeric and centromeric regions [26]. To include more information and better reflect the original siRNA situation, 931,924 siRNA loci containing multi-reads (≤1000 locations) were used for further analysis, with more than 90% of the siRNA loci predominantly producing 24-nt siRNAs and 3% and 1% of the loci being enriched with 21- and 22-nt siRNAs, respectively (Additional file 3: Table S2).

To statistically evaluate the association of the 21-, 22- and 24-nt siRNA loci with genic, intergenic and repetitive regions, we compared the observed occurrence with the simulated occurrence of 21-, 22- and 24-nt siRNA loci in association with the three genomic region types (Mehtods). The 21-nt siRNA loci showed a significantly high degree of association with genic regions (64.6% observed vs. 10.7% simulated) and a low with intergenic regions (17.6% observed vs. 75.4% simulated); the 22-nt siRNA loci had a relatively high degree of association with repetitive regions (92% observed vs. 72% simulated) but low with intergenic regions (3.8% observed vs. 17.6% simulated). In contrast, the 24-nt siRNA loci showed no obvious enriched tendency within any genomic region (Additional file 4: Table S3).

**Figure 2:**
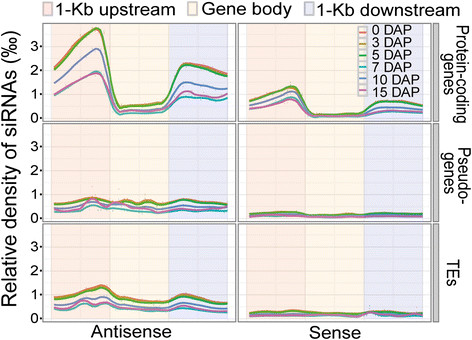
**Genic distribution of 24**-**nt siRNAs.** Relative densities of 24-nt siRNAs in 5′ 1-kb upstream regions, gene body regions and 3′ 1-kb downstream regions were calculated separately on sense (right lane) and antisense (left lane) strands for 0-, 3-, 5-DAP kernels and 7-, 10-, 15-DAP endosperms in protein-coding genes, pseudo-genes and TEs, respectively. The results indicate 24 nt-siRNAs were highly enriched in the 1-kb upstream and downstream regions in antisense strands, and the density of 24-nt siRNAs in the 10-DAP endosperm were much higher than those in other two stages of endosperm. Orange-red: 5′ 1-kb upstream regions, Orange: gene body regions, bluish gray: 3′ 1-kb downstream regions. Red line: 0-DAP kernel, brown line: 3-DAP kernel, green line: 5-DAP kernel, blue line: 7-DAP endosperm, dark blue: 10-DAP endosperm, dark red: 15-DAP endosperm.

Furthermore, we examined the detailed distributions of 24-nt siRNAs by plotting their relative densities along protein-coding genes, transposable elements (TEs) and pseudogenes plus the 1-kb upstream and 1-kb downstream regions. During the density calculation, the contribution of a multi-read to a locus was divided by its mappable location number to minimize the potential bias. Interestingly, different patterns of 24-nt siRNA enrichment were observed in different gene classes, genic territories, developmental stages and strands (Figure 2). First, the siRNA density was significantly higher in protein coding genes than in pseudogenes and TEs in terms of the 1-kb flanking regions, and the protein-coding genes themselves exhibited a much greater siRNA density in the 1-kb flanking regions compared with the gene body regions. Second, 24-nt siRNAs were more enriched on the antisense strands than on the sense strands of protein-coding genes. Third, the overall density of 24-nt siRNAs in the kernels was much greater than in the endosperm, and the 24-nt siRNAs in the 10-DAP endosperm were more dense than those in the 7- and 15-DAP endosperms (Figure 2).

### Activation of paternal siRNAs in maize kernels and endosperms

The paternal genome inherited from the sperm is initiated in a coordinated manner upon double fertilization. Supported by the results from the high throughput sequencing and comprehensive SNP information from the comparison of the fully assembled Mo17 and reference B73 genomes [22], we successfully differentiated the parental origin of the siRNA reads for more than 140,000 siRNA loci, enabling us to examine the process of paternal siRNA activation during the early stages of maize kernel development. Because long gaps caused by insufficient reads in the assembled Mo17 genome could lead to inevitable bias, siRNA loci with gaps of longer than 5% of their own length were not considered (74,521 siRNA loci remaining). To minimize the influence of ambiguous mapping, siRNA loci containing paternal reads in un-pollinated 0-DAP kernels or with low expression level (Materials and Methods) were removed, and 39,126 siRNA loci were retained for further analysis.

**Figure 3:**
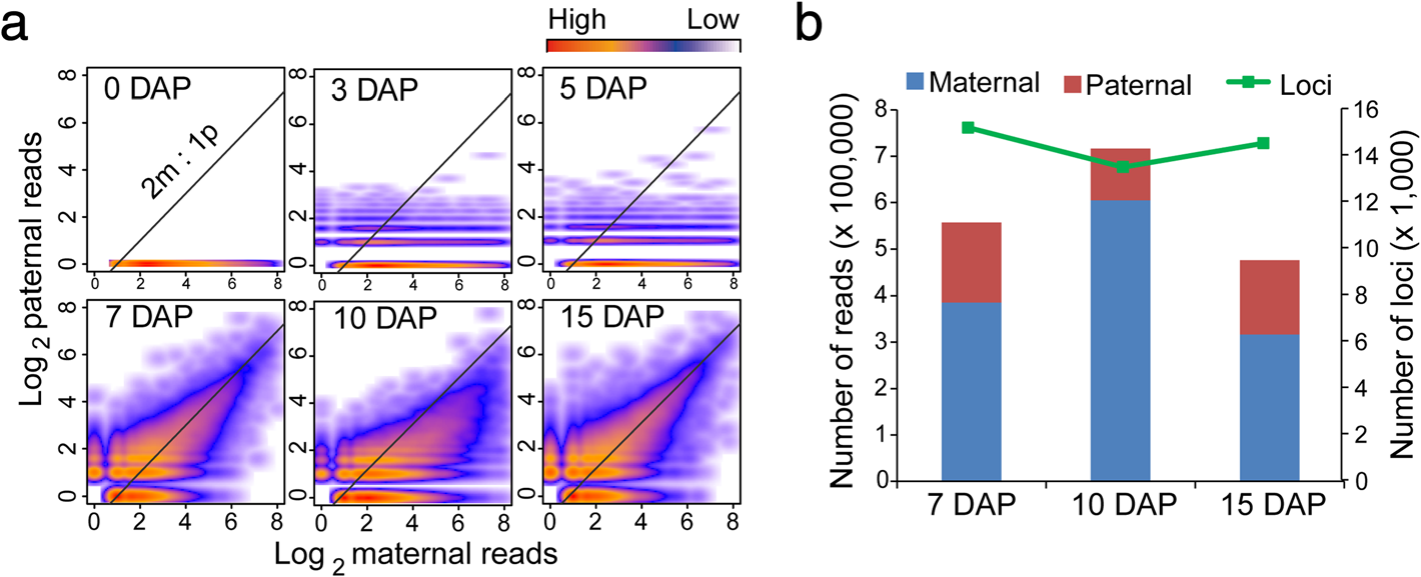
**Activation of paternal siRNA loci in developing maize kernels and endosperms. a**. Plot of the maternal reads versus the paternal reads of siRNA loci during different developmental stages. Paternally derived siRNAs were observed in the 3- and 5-DAP kernels, albeit at low abundance. The ratio of parental siRNA loci expression is likely to reach 2 m:1p in the 7-DAP endosperm. The expression level of paternally expressed siRNAs (y axis) and maternally expressed siRNAs (x axis) are represented by the log_2_-transformed sum of the paternally- and maternally-derived reads in the reciprocal crosses, respectively. Blue (low), yellow (medium) and red (high) represent the relative densities of the siRNA loci at different expression levels. **b**. Number of siRNA loci and counts of parental reads derived from the siRNA loci during the three developmental stages of the endosperm. DAP: days after pollination.

By plotting the paternal versus maternal expression of the remaining siRNA loci, we observed a gradual activation pattern of paternal siRNAs after fertilization in 3- and 5-DAP kernels in terms of the numbers of expressed paternal siRNA loci and their increased expression levels (Figure 3A). From 0 to 3 DAP and from 3 to 5 DAP, 1,165 loci with 3,112 paternal siRNAs and 1,372 loci with 3,956 paternal siRNAs, respectively, were activated. The relatively small proportion of activated siRNA loci and low expression levels were most likely caused by a large proportion of maternal tissue. Even so, we can still conclude that the paternal siRNAs are initiated earlier in maize kernels than previously reported in *Arabidopsis* seeds, in which, the paternal siRNAs remained silent until 5 DAP [27]. However, this predominantly maternally biased expression pattern of siRNAs in the kernels may not necessarily indicate a delayed activation of siRNA paternal alleles compared with the maternal alleles because more than 99% of the loci that were identified in the endosperm samples were also detected in the kernel samples.

In the 7-DAP endosperm, the parental siRNAs reached a normal dosage ratio of 2 m:1p, implying an early full paternal siRNA loci activation and a balanced siRNA biogenesis between the two parental genomes (Figure 3a and b). Interestingly, the proportion of parental siRNAs was the same as that of the protein-coding genes in the 7-DAP endosperm, consistent with the 2 m:1p parental genome composition [22]. Compared with the 1,372 paternally activated loci with 3,956 paternal reads in the 5-DAP kernels, 15,172 loci with 171,235 paternal reads, 13,381 loci with 111,678 paternal reads and 14,321 loci with 159,329 paternal reads were found in the 7-, 10- and 15-DAP endosperms, respectively (Figure 3b). The large difference in paternal siRNA expression between the 5-DAP kernels and the 7-DAP endosperm also supports our speculation that the paternal alleles are likely initiated earlier in the endosperm; rather than an abrupt siRNA burst from the paternal genome within only 2 days, because these alleles were hardly sampled in the kernels due to the high proportions of maternal tissues around the endosperm and embryo. In the 10-DAP endosperm, we observed a global maternal bias of siRNA expression deviating from 2 m:1p, consistent with the report in *Arabidopsis* endosperm in which an abrupt increase of maternal Pol IV-dependent siRNAs occurred from 4 to 6 DAP, followed by reduced or even undetectable maternal p4-siRNA expression levels during the later developmental stages [17].

### Dynamic expression of imprinted siRNA loci

**Figure 4:**
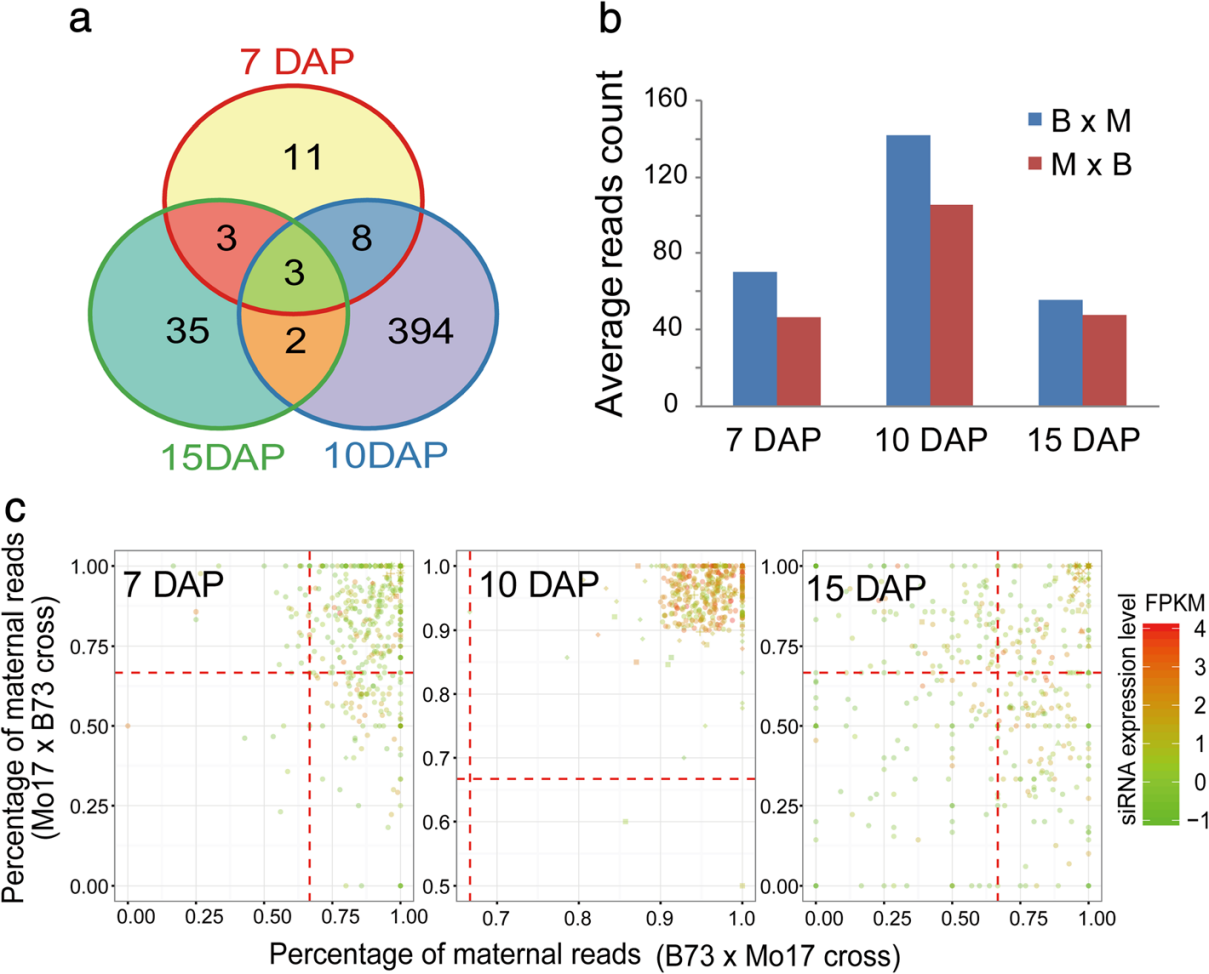
**Identification of imprinted genes in the maize endosperm. a**. Number of maternally expressed siRNA loci (MESL) that were identified in the 7-, 10- and 15-DAP endosperm. **b**. Average read count of 10-DAP-specific imprinted siRNA loci in the three developing endosperm samples of the B73 and Mo17 reciprocal crosses. **c**. Dynamic imprinting status of imprinted siRNA loci during endosperm development. Spots clustered at the upper-right corners at 10 DAP have more than 90% maternal reads (MESLs). DAP: days after pollination.

Based on the parental expression plotting of the siRNA loci, we observed a subset of siRNAs that were expressed maternal-only or paternal-only in a parent-of-origin pattern (Figure 3A), and this type of biased expression of siRNAs has been previously reported in rice and *Arabidopsis*[17],[18]. To avoid the influence of maternal tissue in the maize kernels, we decided to identify imprinted siRNA loci only in 7-, 10- and 15-DAP pure endosperms using the following procedures. First, a set of approximately 140,000 identified SNP-containing siRNA loci were prescreened using two criteria: the locus on the Mo17 genome must not contain a gap ≥ 5% of its length, and there must be no paternally derived reads mapped in the 0-DAP kernel. Then, the χ^2^ test was performed to examine whether the observed ratios of the maternally versus paternally derived reads significantly deviated from the normal ratio of 2 m:1p in each cross. Furthermore, a more stringent cutoff was employed to precisely define the MESL and paternally expressed siRNA loci (PESL), respectively, namely at least 90% of the maternal reads or 70% of the paternal reads from the total SNP-associated reads (with a minimum of 20 reads) mapped to a locus in both reciprocal crosses. At the significance level of *p* = 0.01, the parental expression ratio of 2,220, 2,451 and 2,136 siRNA loci were detected to have a significant deviation from 2 m:1p in both of the reciprocal crosses, and among which, 59, 1,188 and 110 loci exhibited a parent-of-origin pattern at 7, 10 and 15 DAP, respectively. Finally, a set of 460 candidate imprinted siRNA loci were retained in the three endosperm samples based on the reads count cutoff, including 456 MESL and only 4 PESL. Except for three and thirteen loci that were simultaneously imprinted at three and two stages, respectively, the majority of the MESL (444 of the 456) were stage-specific, with 394 (88.7%) occurring only at 10 DAP (Figure 4a and Additional file 5: Table S5). By plotting the maternal versus the paternal expression of all the imprinted siRNA loci using SNP-containing reads in the 7-, 10- and 15-DAP endosperms, we found that the expression levels of the 10-DAP-specific MESL peaked at 10 DAP and decreased at 7 and 15 DAP, and their parental expression ratio also dynamically changed: at 7-DAP, these MESL already exhibited a maternally biased expression pattern, and their parental expression ratios were significantly deviated from the expected 2 m:1p ratio (χ^2^ test, *p* ≤ 0.01) but failed to pass the ratio cutoff (90%). Later, at 10 DAP, their maternal bias was more deviated due to the elevated siRNA expression of the maternal alleles and satisfied our imprinting screen criteria; However, these MESL changed to be biallelically expressed at 15 DAP (Figure 4B and C). Conclusively, the transient imprinting status of the 10-DAP-specific MESL was likely attributed to the dynamically changed expression level and parental dosage ratio at 10 DAP compared with 7 and 15 DAP.

### Imprinted genes might be influenced by the adjacent imprinted siRNA loci

**Figure 5:**
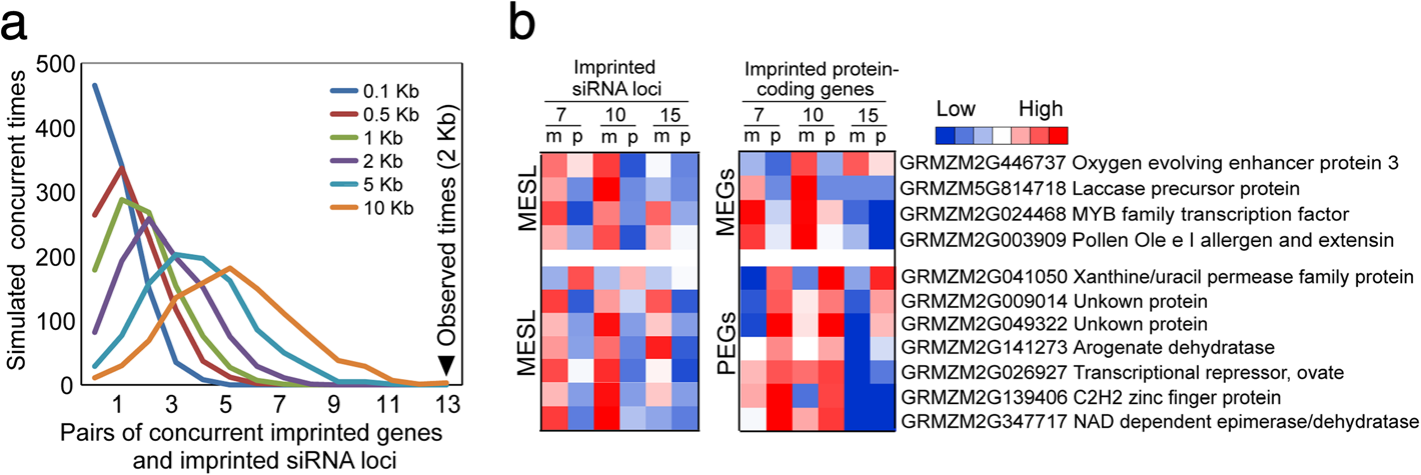
**Correlation of imprinted siRNA loci and imprinted genes in maize endosperm. a**. Simulated concurrence of imprinted genes and imprinted siRNA loci based on 0.1 kb, 0.5 kb, 1 kb, 2 kb, 5 kb and 10 kb. The observed number of concurrent imprinted genes and imprinted siRNAs was significantly higher than that derived from 1000-time simulation according to the hypergeometric distribution test. The peaks in each line indicate the biggest time of simulated concurrent imprinted genes and imprinted siRNAs in terms of different length of flanking regions. **b**. Parental expression heat map of 11 pairs of imprinted genes and imprinted siRNAs in three developing endosperm based on 2-kb criteria (expression data of two imprinted genes are unavailable).

The stage-dependent imprinting pattern of the 10-DAP-specific MESL is reminiscent of the enrichment of 10-DAP-specific MEGs reported by Xin et al. [22] using the same set of maize endosperm smaples. To examine whether the expression of imprinted genes were potentially influenced by their adjacent imprinted siRNA loci, we specifically examined imprinted siRNAs within the 2-kb flanking regions of the 371 imprinted genes combined from the previous three studies [22],[28],[29]. Of the 371 imprinted genes, 357 were associated with a siRNA locus, including 298 that harbored SNP-containing siRNA loci. A total of 166 imprinted genes were further excluded because their harbored siRNA loci contained a gap longer than 5% of its length on the Mo17 genome or contained paternal reads at 0 DAP. Of the remaining 132 siRNA loci, only 31 siRNA loci adjacent to 29 imprinted genes had a minimum of 20 siRNA reads and were included in the final list for the determination of their imprinting status. Finally, 13 of the 29 imprinted genes (13 paternally expressed genes (PEGs) and 16 MEGs) contained strictly defined imprinted siRNA loci located within their 2-kb flanking regions. This fraction (13/29) was considered a significant co-occurrence of imprinted genes and imprinted siRNA loci based on the simulation analysis (Figure 5a). Specifically, we randomly placed the same amount of imprinted genes onto the genome and counted how many imprinted siRNA loci were located within their 0.1, 0.5-, 1-, 2-, 5- and 10-kb flanking regions. The simulation was performed 1,000 times to generate a series of empirical distributions of the numbers of paired imprinted genes and imprinted siRNA loci. The distributions indicated that the chance of 13 co-occurrence of imprinted genes and imprinted siRNA loci was significantly higher than the expected numbers generated by the simulations, examined by the hypergeometric distribution test (*p* = 2.122 × 10^−6^) (Figure 5a).

Among the thirteen pairs of associated imprinted protein-coding genes and imprinted siRNA loci, five pairs were MEGs and MESL, one pair was a PEG and PESL, and the remaining seven pairs were PEGs and MESL (Figure 5b). The last seven pairs were potentially interesting due to their opposite parental expression patterns because maternally derived siRNAs might be involved in the epigenetic silencing of the maternal alleles in *cis*, causing the paternal allele-specific expression of their adjacent imprinted genes. For example, the PEG GRMZM2G139406, encoding a C2H2-type zinc finger protein was highly expressed in the 7-DAP endosperm, but the expression level decreased at 10 DAP and undetectable at 15 DAP, harboring a MESL exhibiting an opposite imprinting status in the three stages (Additional file 6: Figure S2).

### SiRNAs are likely to participate in nutrient uptake and allocation in the developing maize endosperm

**Figure 6:**
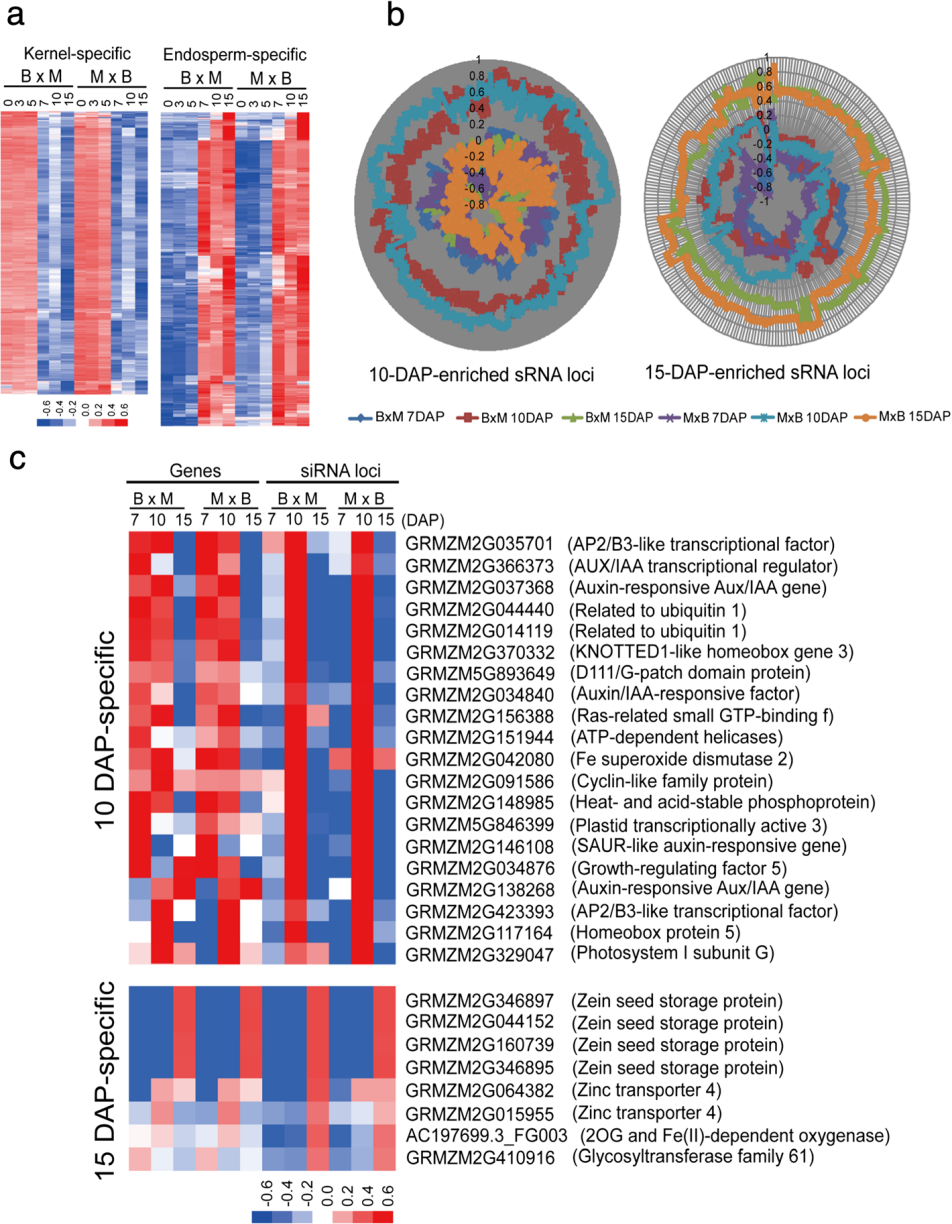
**Functional enrichment of genes harboring 10**-**DAP**-**specific and 15**-**DAP**-**specific siRNA loci within 2**-**kb flanking regions. a**. Expression heat map of kernel-specific and endosperm-specific siRNA loci. 453 kernel-specific and 729 endosperm-specific siRNA loci were identified based on a cutoff of a five-fold difference in average siRNA abundance among the three kernel stages (0, 3, 5 DAP) and three endosperm stages (7, 10, 15 DAP), containing at least 1,000 reads combined from the 12 samples, red indicates high expression level, blue indicates low expression level. **b**. Expression analysis of 10-DAP-specific and 15-DAP-specific siRNA loci in the B73 and Mo17 reciprocal crosses. The siRNA abundance in 7-, 10- and 15-DAP endosperm were first normalized and then clustered based on a five-fold difference in average siRNA abundance among different endosperm developmental stages in both reciprocal crosses, and 776 and 270 stage-enriched siRNA loci in the 10- and 15-DAP endosperms were identified, respectively. **c**. Heatmap of 10-DAP and 15-DAP specifically expressed siRNA loci and their associated genes enriched in the GO analysis. The color scale in blue (low), while (medium), and red (high) represents the relative expression level of maternal or paternal alleles, “M” represents Mo17, “B” represents B73.DAP: days after pollination.

Genomic distribution of siRNA loci was preferentially enriched in high-density gene and chromosome ends regions and there was a higher density of siRNAs flanking protein-coding genes compared with TEs and pseudogenes (Figures 1 and 2). To better understand the potential functions of siRNAs in maize kernels and endosperms, we classified these siRNA loci as kernel-specific and endosperm-specific based on a cutoff of a five-fold difference in average siRNA abundance among the three kernel stages (0, 3, 5 DAP) and three endosperm stages (7, 10, 15 DAP). We identified 453 kernel-specific and 729 endosperm-specific loci from 51,071 siRNA loci containing at least 1,000 reads combined from the 12 samples. A hierarchical clustering of these siRNA loci showed a higher degree of expression changes in the endosperm compared with that in the kernels which may indicates a more dynamic activity of siRNA loci in the developing endosperm (Figure 6a). A Blast2GO analysis showed that 316 genes harboring endosperm-specific siRNA loci within their 2-kb flanking regions were predominantly enriched in the “RNA splicing via transesterification reactions”, “DNA recombination” and “protein-DNA complex assembly” terms (Additional file 7: Table S4). Interestingly, these genes were also enriched in the “regulation of telomere maintenance” category, consistent with the dense distribution of siRNAs in the maize chromosome ends (Figure 1). In contrast, the 257 genes harboring kernel-specific siRNA loci were significantly enriched in the “response to cytokinin stimulus” and “response to organic nitrogen” GO functional categories (Additional file 7: Table S4).

Furthermore, we identified 27, 776 and 270 stage-enriched siRNA loci in the 7-, 10- and 15-DAP endosperms, respectively, using the same criteria as above (Figure 6b). A total of 12, 551 and 103 protein-coding genes, respectively, contained these corresponding stage-specific siRNA loci in their 2-kb flanking regions. Genes harboring 10-DAP-specific siRNA loci showed a significant enrichment in the “response to auxin stimulus”, “response to brassinosteroid stimulus” and “regulation of gene expression” groups (Figure 6C and Additional file 7: Table S4). This result is consistent with our previous observation in the RNA-Seq data in which 20 genes encoding auxin responsive factor (ARF) showed elevated expression at 10 DAP, followed by decreased expression at 15 DAP [22]. This result is also coincident with the report that the IAA concentration abruptly increased in 10-DAP endosperm [30] and then decreased at approximately 15 DAP [31],[32].

In contrast, genes harboring 15-DAP-specific siRNA loci were enriched in the “nutrient reservoir activity”, “protein localization to vacuole” and “secondary metabolite biosynthetic process” categories, including four members of the zein storage protein family (Additional file 7: Table S4). Interestingly, this result is consistent with the observation that 15 DAP is the stage of rapid fresh weight increase and the biosynthesis of zein proteins and starches [23]. Although the exact mechanism of how these stage-specific siRNA loci are involved in the hormone response and nutrient accumulation remains unclear, our data indicates that siRNA expression may correspond to specific cellular or biochemical processes that are essential for endosperm or kernel development.

## Discussion

### Paternal siRNA loci are activated during the early stages of maize seed development

In angiosperms, the activation of the parental mRNA and small RNA transcriptomes after fertilization in seeds remains ambiguous, although some recent studies have indicated that paternal genome activation lagged behind that of maternal genome [7]-[9],[17],[27]. However, other studies have suggested that the parental genomes might contribute equally during the early stages in the zygotes [10]-[12].

In this study, the data enabled us to survey the dynamics of the allelic expression of endogenous siRNAs, i.e. the timing and proportion of parental siRNA loci expression at 3 and 5 DAP during maize seed development, and consistent with the report in *Arabidopsis* that siRNAs are mostly maternal-predominant in maize kernels before 5 DAP, [17],[27]. However, our data also indicated that the transcriptional activation of the paternal siRNA loci in the maize kernel occurred immediately upon fertilization, as demonstrated by the presence of paternal siRNA reads in 3 DAP and 5 DAP kernels (Figure 2), which indicates that siRNA biosynthesis might be differentially regulated in the persistent endosperm of maize and the ephemeral endosperm of *Arabidopsis*, where paternal siRNAs remain largely silent during the first 5 DAP [17],[18]. Due to delayed development and smaller proportions of the embryo compared with the endosperm in maize during the first few DAP [8], we speculated that the activation of paternal siRNAs mainly occurred in the endosperm before 5 DAP. Additionally, paternal siRNA activation might have been completely achieved at least by 7 DAP based on 2 m:1p expression ratio at this developmental stage (Figure 2), leading to the balanced siRNA landscapes of the two parental genomes. The significant difference of the paternal siRNA expression between the 5-DAP kernel (1,372 loci with 3,956 reads) and 7-DAP endosperm (15,172 loci with 171,235 reads) was not likely due to an extremely fast activation of the paternal siRNA alleles within a short period of only 1 or 2 days, but rather, the high proportion of maternal tissues (e.g., pericarp and nucellus) in the kernels largely reducing the detection probability of paternal siRNAs originating from the endosperm. This activation process might be supported by the reprogramming of CHH methylation of the paternal genome that is subject to hypomethylation in the sperm cells before fertilization [21] and later, shared a similar CHH methylation status with the maternal genome in the endosperm after fertilization [33].

Furthermore, siRNA loci and protein-coding genes shared a similar pattern of the paternal allele activation namely, both of their paternally activated alleles were detected in very low read abundances in the 3- and 5-DAP kernels, and then reached the normal ratio of 2 m:1p in the 7-DAP endosperm [22]. However, we failed to detect any concurrent relationship between the paternally activated siRNA loci and protein-coding genes in terms of their 1-, 2- and 5-kb adjacent regions. This result might be either due to an insufficient coverage of the mRNA and siRNA transcriptomes because of the small fractions of endosperm and embryo tissues in the entire kernel or the initial transcriptional activation of the paternal alleles randomly occurring on genomic loci, as the paternally activated alleles in the B73 and Mo17 reciprocal crosses showed a very small overlapping fraction [22].

### A subset of imprinted siRNAs are potentially responsible for imprinted gene expression

Due to the crucial role of 24-nt siRNAs in the RNA-directed DNA methylation (RdDM) pathway, the dynamic change in siRNA expression in developing maize endosperm may reflect the distinct activity of RdDM pathways before and after 7 DAP in the maize endosperm, consistent with the previous report in *Arabidopsis* that the RdDM components were expressed quite low during the early endosperm developmental stages and substantially increased to high levels during the later stages [34]. Therefore, we hypothesize that the immediately activated paternal siRNAs upon fertilization [21], gradually reached the balanced parental expression dosage at some point before 7 DAP in the endosperm, and then invoked the *de novo* methylation assisted by co-expression of RdDM components. This result may explain why the global CHH methylation levels between the maternal and paternal alleles were almost the same in the *Arabidopsis* endosperm [33].

Imprinted siRNA loci may contribute to the formation of the parent-specific DMRs, which is one of the major mechanisms resulting in the imprinted expression of neighboring genes [14]-[16]. Until now, several lines of evidences have indicated the important role of siRNAs in genomic imprinting. First, the maternally expressed *SDC* and *MOP9.5* involved siRNAs that silenced the paternal alleles through the RdDM pathway after fertilization in the *Arabidopsis* endosperm [20]. Second, studies of 12 transposable elements surrounding MEGs showed that 24-nt siRNAs targeted the paternal alleles of these MEGs within 2-kb flanking regions, resulting in the paternally specific CHH hypermethylation and the corresponding paternal-specific silencing [21]. Third, 4 out of 31 imprinted siRNA loci were enriched in DMRs in rice and overlapped with known imprinted genes in the 7- to 8-DAP endosperm of *Nipponbare* and *Kitaake* reciprocal crosses with an opposite imprinting status [18]. Our examination of the association probability between the imprinted siRNA loci and imprinted genes in maize indicated that the imprinted siRNA loci are likely positionally associated with imprinted genes based on the comparison of the observed and simulated possibilities of co-occurrence (Figure 5). Moreover, seven out of thirteen pairs of imprinted genes and imprinted siRNA loci were expressed from opposite parental alleles, indicating that imprinted siRNA may function in recruiting allele-specific *de novo* methylation to silence the corresponding alleles. However, we also found six pairs of imprinted genes and imprinted siRNA loci expressed from the same parent allele. One possible explanation is that some other mechanism may be involved in the regulation of these imprinted genes besides siRNA-mediated *de novo* methylation. For example, the paternally expressed of *PHE1* in *Arabidopsis* was due to hypermethylattion of tandem repeats downstream of the paternal allele, which prohibited the binding of Polycomb Repressive Complex 2 (PRC2) to the paternal *PHE1* allele to form the silencing H3K27me3 histone modification [35],[36].

### Dynamic parental siRNA expression is correlated with key biological events during the development of maize endosperm

The parental expression of siRNA loci showed maternal bias in the 10-DAP endosperm, deviating from the normal ratio of 2 m:1p at 7 DAP and 15 DAP (Figure 3a). Correspondingly, 89% of the imprinted siRNA loci exhibiting strong maternal expression were specific to the 10-DAP stage, as a result of the elevated expression level of maternal siRNAs compared with that at 7 and 15 DAP (Figure 4C). This result is consistent with the previous report in *Arabidopsis* that the maternal allelic expression of certain classes of p4-siRNAs peaked at 4–6 DAP and then gradually decreased to reduced or even undetectable levels during later stages [17]. However, the biological function of p4-siRNAs in the endosperm remains ambiguous because even the mutant *nrpd1*, containing mutations in the largest subunit of PolIV which regulates the biosynthesis of most siRNAs, showed no obvious morphological defects in *Arabidopsis*.

However, a GO analysis of genes harboring siRNA loci within their 2-kb flanking regions indicated that the biogenesis of siRNAs might be closely related to the key developmental events of the endosperm. Overall, the GO terms of genes harboring adjacent siRNA loci indicated that siRNAs most likely influence endosperm development at not only the transcriptional level but also the post-transcriptional level. In addition, the 10-DAP endosperm-enriched siRNA loci showing strong maternally biased expression specifically attracted our attention because 10 DAP is an important stage that coincides with the rapid increase in auxin accumulation and the onset of the starch and particular zein protein biosynthesis in the maize endosperm [23],[30]. Moreover, the maize mutant de*-*B18* (*defective endosperm*-*B18*), with decreased auxin levels, had less dry matter accumulation in the seeds [37]. Interestingly, 10-DAP-specific siRNA-associated genes were significantly enriched in the “response to auxin stimulus” category, and their GO terms also included “response to brassinosteroids (BRs) stimulus”, reminiscent of their potential function in seed development in which BRs regulate the nutrient assimilation from source to sink and the deficient or insensitive BR mutants produce small-sized seeds in rice [38]-[41]. Therefore, although the exact molecular mechanisms still remain unknown, our results indicate that the dynamic activity of siRNA loci might play a crucial role in nutrient uptake and allocation during endosperm development.

## Conclusions

SRNAs are likely to play a crucial role in the maintenance of genome stability in the maize endosperm, and GO term analyses of siRNA associated genes suggests they are also involved in the regulation of nutrient uptake and storage. Systematic analysis of parental siRNA expression indicates a subset of siRNAs subject to allele specific expression in a parent-of-origin manner, and this imprinted expression exhibited dynamic patterns in developing maize endosperm. Additionally, the imprinted expression of sRNAs could be one of causes for gene imprinting based on the association analysis.

## Methods

### Plant materials

The maize B73 and Mo17 inbred lines were grown under greenhouse conditions. The reciprocal crosses and self-pollination were performed as follows. First, the ears were bagged before the growth of silk. Then, when the silks grew to approximately 2 to 3 cm, the ears were cut 2 cm from the top, and the tassels were bagged. The pollination was conducted the next day using the corresponding pollen. The un-pollinated kernels (0-DAP) and the kernels from the reciprocal crosses of B73 and Mo17 at 3, 5, 7, 10 and 15 DAP were harvested. The endosperm tissues from the 7-, 10- and 15-DAP kernels were manually isolated. The endosperm and kernels were harvested from three different ears as three biological replicates and were immediately frozen in liquid nitrogen.

### Small RNA library construction and sequencing

The total RNA was isolated from the 36 (12 samples × 3 replicates) groups of plant material using the TRIzol reagent according to the manufacturer’s instructions. The RNA samples were sent to the Beijing Genomics Institute (BGI) at Shenzhen to isolate small RNAs. Before the library construction, the RNA quality of the 36 total RNA samples was examined using an Agilent 2100 Bioanalyzer. The three biological replicates for each sample were all of high quality and thus were combined to construct the small RNA libraries using the BGI’s standard protocol. Small RNA samples with ligated adaptors were sequenced by Illumina Solexa to generate 36-nt single-end reads. The adaptors were trimmed from the 36-nt reads by BGI. This whole Solexa sequence has been deposited at GenBank under the accession GSE52726 http://www.ncbi.nlm.nih.gov/geo/query/acc.cgi?acc=GSE52726.

### Genic distribution of 24-nt siRNAs

First, gene body and their 1-kb upstream and 1-kb downstream regions were individually divided into 20 equal-sized bins. Then, the siRNAs perfectly mapped to each bin of a gene territory were counted for sense and antisense strands separately. Finally, to plot relative density of 24 nt-siRNAs in gene body and its upstream and downstream regions, their proportions in each bin were normalized by the total number of mapped reads along all three regions, and next, the 24 nt-siRNA densities were averaged by the number of protein-coding genes, pseudogenes or TEs, respectively.

Simulation analysis were performed as follows: the same number of observed 21-nt, 22-nt and 24-nt siRNA loci were randomly placed onto maize genome using ShuffleBed program in the BEDTool, respectively, and counted how many times these siRNA loci were located in genic, intergenic and repetitive regions. This simulation analysis was performed for 1,000 times to generate a series of empirical distributions, and based on which, statistical significance between siRNA and genomic regions was calculated, respectively.

### Identification of siRNA loci and siRNA expression calculation

We used a previously described method to define the siRNA loci. First, the combined reads from the 12 small RNA samples were mapped together to the B73 reference genome by Bowtie1, and only the perfectly matched reads were used to identify the siRNA loci. Then, the overlapping reads were merged to form siRNA “islands” that contained at least three reads. Finally, the neighboring islands within 100 bp were further merged as one siRNA locus. To minimize the artifacts of ambiguous mapping, we retained only those siRNA loci with more than three reads in at least two libraries. When calculating the siRNA expression level of a locus, the contribution of a multiply mapped read to a locus was divided by the number of the read’s mapped locations.

## Abbreviations

siRNA: small interfering RNA

DAP: days after pollination

2 m:1p: 2-maternal vs. 1-paternal

DMRs: differential methylation regions

*SDC*: *SUPPRESSOR OF drm1 drm2 drm3*

MEGs: maternally expressed genes

PEGs: paternally expressed genes

GO: gene ontology

MESL: maternally expressed siRNA loci

nt: nucleotides

TEs: transposable elements

PESL: paternally expressed siRNA loci

## Competing interests

The authors declare that they have no competing interests.

## Authors’ contributions

ZN, XW, QS conceived the project. MX, collected the plant materials. MX, RY, YY, HP and MC analyzed data. MX, XW and NZ. wrote the manuscript. All authors’ read and approved the final manuscript.

## Additional files

## Supplementary Material

Additional file 1: Figure S1. Small RNA size distribution with Solexa high throughput sequencing. Click here for file

Additional file 2: Table S1. Small RNA mapping profile summary. Click here for file

Additional file 3: Table S2. Description of identified small RNA clusters based on different criteria. Click here for file

Additional file 4: Table S3. Differential distribution of 21-, 22- and 24-mer clusters in gene loci, repeats and intergenic regions. Click here for file

Additional file 5: Table S5. Allele-specific expression data of the 460 imprinted small RNA clusters. Click here for file

Additional file 6: Figure S2. Parent-of-origin expression patterns of GRMZM2G139406 and its harboring MESL, Mega21192.9. Click here for file

Additional file 7: Table S4. GO category enrichments of kernel-specific siRNA-associated genes, endosperm-specific siRNA-associated genes and 10- and 15-DAP specific siRNA-associated genes. Click here for file
