# Supplementary figures and images for: Dynamic parent-of-origin effects on small interfering RNA expression in the developing maize endosperm
Source: BMC Plant Biol. 2014 Jul 24;14:192. doi: 10.1186/s12870-014-0192-8 (PMC4222485; doi:10.1186/s12870-014-0192-8)

## small RNA size distribution

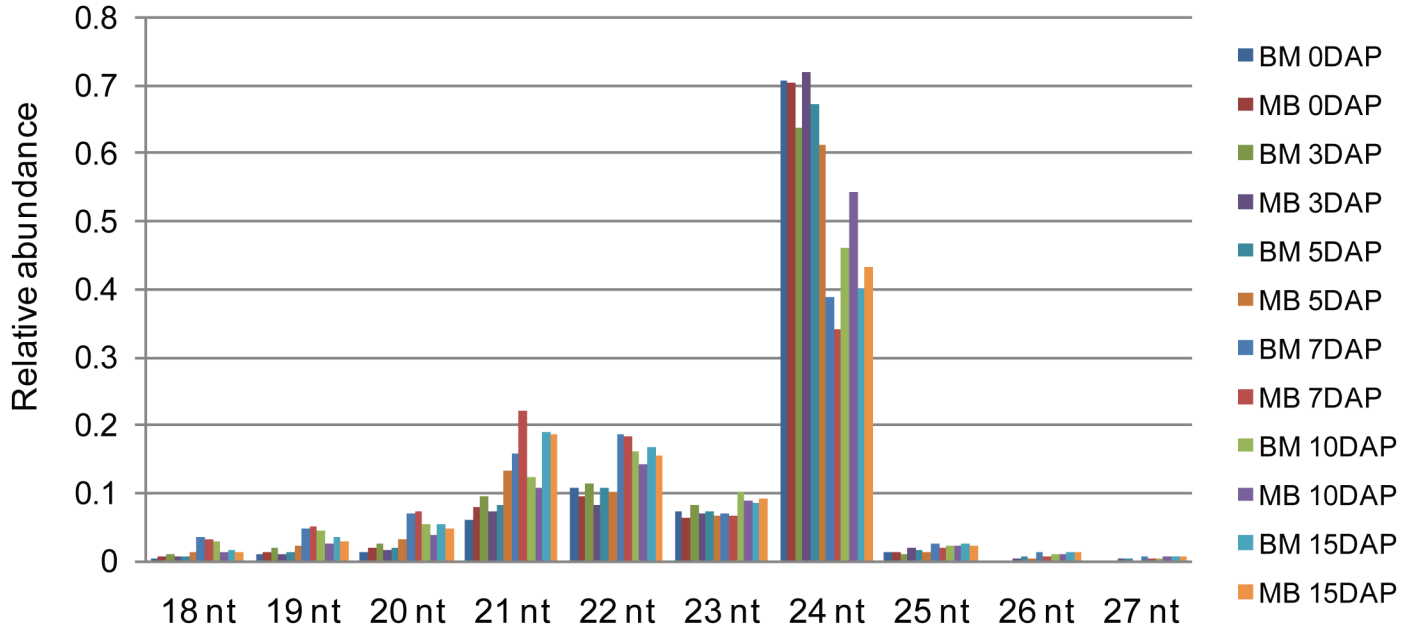

Supplement: Additional file 1: Figure S1. — Small RNA size distribution with Solexa high throughput sequencing. [file s12870-014-0192-8-S1.pdf]

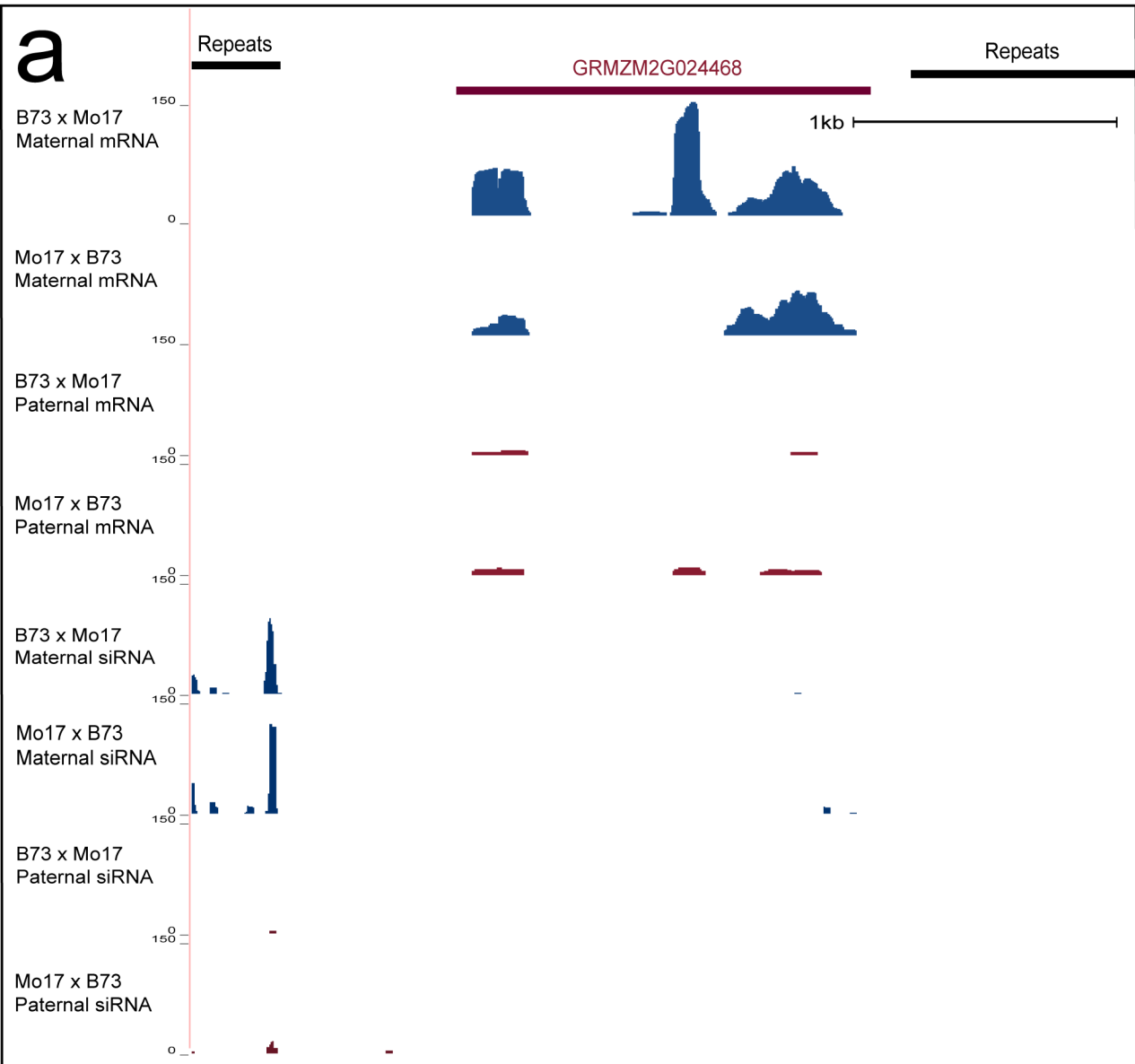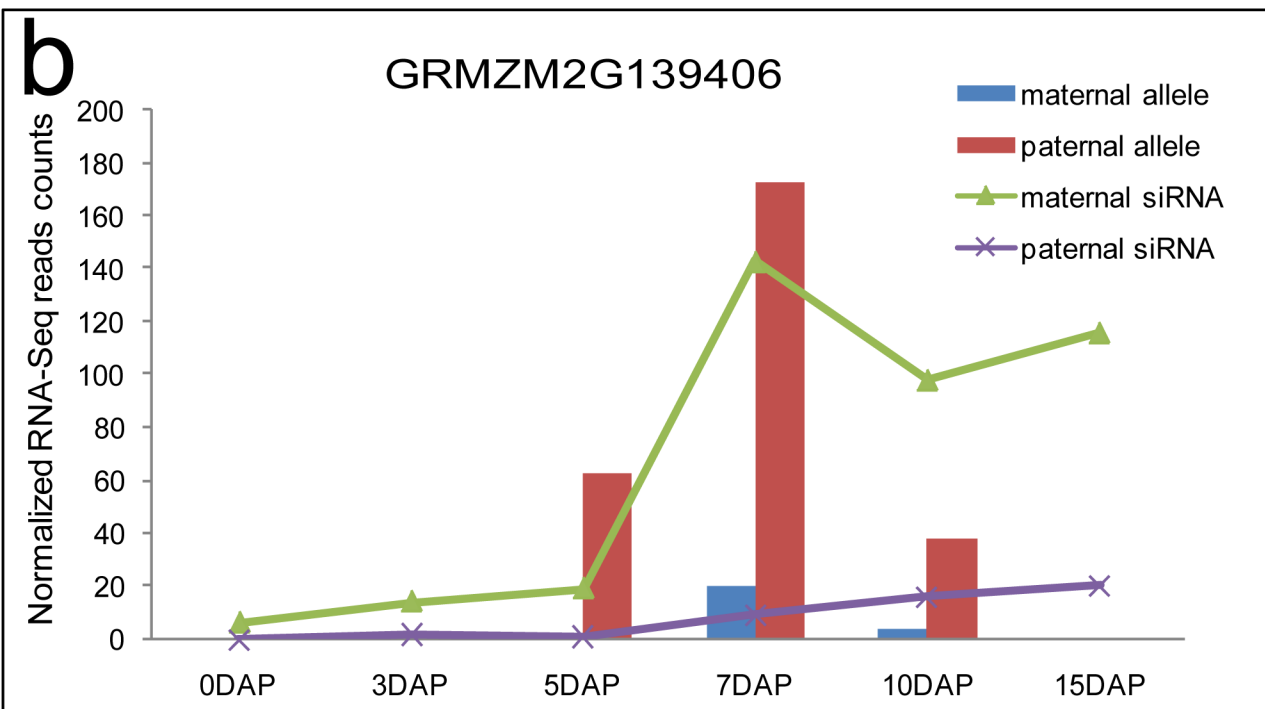

Supplement: Additional file 6: Figure S2. — Parent-of-origin expression patterns of GRMZM2G139406 and its harboring MESL, Mega21192.9. [file s12870-014-0192-8-S6.pdf]
